# Growing Old in a Transnational Setting: Investigating Perceptions of Ageing and Changing Filial Ties Among Older Indians in Saskatoon

**DOI:** 10.1007/s10823-021-09428-w

**Published:** 2021-04-28

**Authors:** Jagriti Gangopadhyay

**Affiliations:** Manipal Centre for Humanities, Manipal Academy of Higher Education (MAHE), Manipal, Karnataka 576104 India

**Keywords:** Successful Ageing, Perceptions of ageing, Intergenerational relationships, Transnationalism, Gender, Older Indians, Saskatoon, Qualitative

## Abstract

Numerous studies have examined the experience of growing old in a transnational context among Indians. However, in most of these studies, the older adults had immigrated as senior citizens to be with their adult children. Indians who have grown old in transnational settings have not been examined in detail in the gerontological scholarship. Adopting a cross-cultural lens, the present study focusses on perceptions of ageing among older Indians who have grown old in the city of Saskatoon. The study demonstrates how these older Indians refute the Successful Ageing model and accept their physical weaknesses in their course of ageing. Additionally, the study also examines how caregiving arrangements and intergenerational relationships are shaped among these older Indians and their adult children, in a transnational city, such as Saskatoon. Finally, the study highlights how later life gender roles are constructed in a transnational backdrop.

## Background

Several studies have analyzed the subjective experience of growing old among older South Asians in transnational contexts (Lamb, 2002, 2009; Treas & Mazumdar, 2002; Kalavar & Van-Willigen, 2005; Lunt, 2009; Patel, 2011; Sudha, 2014; Baldassar & Merla, 2014; Nare, 2017; De Silva, 2018; Hromadzic & Palmberger, 2018). In particular, these studies have indicated how the transnational setting plays an essential role in shaping caregiving arrangements, intergenerational relationships and network ties among older Indian immigrants (Lamb, 2002, 2009; Treas & Majumdar, 2002; Kalavar & Van-Willigen, 2005; Lunt, 2009; Patel, 2011; Sudha, 2014; Nare, 2017). Though these studies have demonstrated significant concerns such as loneliness, cultural adjustments and everyday struggles of South Asian immigrant older parents, nonetheless, the core focus of these studies were older adults who immigrated to live with their adult children after they had retired or experienced widowhood or had some form of health setback.

The present study departs from these studies and focuses on older Indians who had immigrated as young adults for employment reasons and have grown old in a transnational setting. The main research goals of the study are as follows:

- To highlight how physical disabilities form an integral part in shaping the perceptions of ageing among older Indians growing old in a transnational site such as Saskatoon.

- To offer a comparative perspective on intergenerational relationships and caregiving arrangements as maintained by older Indians who live with their adult children and older Indians who do not live with their adult children.

- To shed light on the intersections between ageing, widowhood, gender roles and the transnational site.

By highlighting the ageing experience of a sample population (Older Indians ageing in a transnational backdrop), underrepresented in research, this study is an attempt to expand the existing scholarship in the discipline of social gerontology.

## Theoretical Perspective

The post-modern and biomedical Successful Ageing model has received considerable attention in the gerontological scholarship. The concept of Successful Ageing was introduced by Havighurst in 1961. Initially, scholars Rowe and Kahn (1997) had defined the Successful Ageing perspective as “avoidance of disease and disability, maintenance of high physical and cognitive function, and sustained engagement in social and productive activities” (Rowe & Kahn, 1997: 434). However, this understanding of the model had generated a fair share of criticism (Dillaway & Byrnes, 2009; Lamb, 2014; Katz & Calasanti, 2015; Martinson & Berridge, 2015; Rubenstein & Medeior, 2015). Taking note of this critique, in a more recent article, the scholars, Rowe and Kahn (2015) conceptually expanded the Successful Ageing model. In this article, the authors added that the needs of the contemporary society urge that the “successful ageing of the individual be complemented with a body of theoretical inquiry and empirical research at the level of society” (Rowe & Kahn, 2015: 594). The authors further elaborate on this statement and suggest that to facilitate Successful Ageing, a society requires policies, social institutions, new roles and responsibilities for older adults as well as utilization of the talent and knowledge of the older population for employment or civic engagement.

The Successful Ageing model has been used extensively to understand the experiences of ageing in North American countries (Fischer & Specht; 1999; Lamb, 2014; Molton & Yorkston, 2017). However, there is a shortage of research on the Successful Ageing model and the elderly South Asian diaspora. Additionally, the study population, older Indians growing old in a transnational context has also received limited attention in gerontological scholarship. Addressing these research gaps, the present study demonstrates how older Indians settled in Saskatoon accept their age-related decline and negate the Successful Ageing model. In particular, the study elucidates how older Indians acknowledge the fragility of their bodies and refute the idea of remaining healthy and active. By highlighting the perception of ageing of these older Indians against the Successful Ageing model, the purpose of the study is to illustrate how the South Asian community growing old in transnational settings construct their later lives.

## Methodology and Study Site

The study adopted a qualitative lens to interrogate questions on perceptions of ageing, intergenerational relationships and gender identities of older Indians in a transnational setting. Within the South Asian community, Indians continue to be the largest immigrant group across the world (Conner, 2017). Hence, only older Indians were recruited for this study. Combining convenience and snowball sampling (Babbie, 2012), the study recruited a total of thirty older respondents. Among the thirty respondents, fifteen of them lived with their adult children (Group I), and the other fifteen did not reside with their adult children (Group II). As the researcher was a visiting scholar at the University of Saskatchewan, the mentors of the researcher helped in establishing contacts with the Secretaries and Presidents of different local Indian societies and associations. Using snowball and convenience sampling techniques, the older respondents for the study were identified through the Hindu Society of Saskatchewan, Punjabi Cultural Association of Saskatchewan, Gujarati Samaj of Saskatchewan and Saskatoon Malayalee Association. Additionally, older respondents were also approached for the study through multiple visits to the Shri Lakshmi Narayan Temple located in Saskatoon. Finally, some older respondents who were still working in the University of Saskatchewan and the Saskatoon City Hospital were also contacted to be a part of the study. Most of the older respondents belonged to the professor and doctor community and hence, belonged to the upper-middle class of Saskatoon.

The study was located in Saskatoon as South Asians are the second largest group of immigrants in the city. In recent times, owing to the immigration policies of the province of Saskatchewan, Saskatoon has emerged as an important destination for immigrants. In particular, the Saskatchewan Immigrant Nominee Program (SINP) (2011) sanctions the province’s government to nominate individuals who satisfy the requirements of the local market by advancing their visa procedure (Citizenship and Immigration Canada (CIC), 2011). Recent reports suggest that due to the SINP and with the retirement of Canadian-born workers employment opportunities will open up for immigrants and Saskatoon will continue experiencing high rates of immigration. As per the 2016 Census, within Canada, Saskatoon had the second-highest immigration rate at 1.8%, and the South Asians (1%) were only next to the Chinese (2%) as the ethnic group to immigrate. Additionally, in a recently released report, the Canadian Magazine for Immigration (Feb 2016) listed India (11.4%) as one of the top countries that sent its people to Saskatchewan from 2000 to 2015 (India was second only to Philippines (27%)). Against this backdrop, Saskatoon emerged as an important site to study family ties, caregiving arrangements and intergenerational dynamics within the older Indian immigrant community.

The study relied on the deductive approach (Creswell, 2013) to design the interview instrument: a semi-structured questionnaire. The questionnaire for older adults was developed based on previous gerontological scholarship and was divided into four parts: Part I (Socio-Demographic Profile); Part II (Perceptions of Ageing); Part III (Intergenerational Ties); Part IV (Gender and Ageing). Once the questionnaire was developed, in-depth narrative style interviews were conducted among older respondents from both Group I and Group II.

Similar to the questionnaire for the older respondents, a separate questionnaire was constructed for the co-resident and non-resident adult child. This questionnaire was also developed using the deductive method. Regarding the interviews with the adult child, for the first group (older adults with co-resident adult child), face to face interviews were conducted with the co-habitant adult child. For the second group (older adults with non-resident adult child), the adult child was interviewed through a video call. For the interviews of the adult children, based on the deductive method, a separate questionnaire was constructed. This questionnaire was also constructed through the deductive approach and had three sections. Part I (Socio-demographic Profile); Part II (Relationship with older parents); Part III (Caregiving Arrangement).

For Group I, inclusion criteria was as follows: to be sixty years old, had immigrated to Saskatoon in their early thirties and were living with at least one adult child for the last three years. For Group II, the first two inclusion criteria were similar to the first group, except that they did not live with their adult child. Chronically ill, permanently disabled, childless and older adults who had immigrated in their sixties were excluded from the study. Informed consent was obtained from all the respondents interviewed. For the adult children interviewed through video calls, informed consent was obtained through emails. The interviews were conducted in Hindi, English and Bengali, given the language proficiency of the author in these three languages. Ethical clearance for this study was obtained from the Indian Institute of Technology Gandhinagar (IITGN). For detailed socio-demographic information of the respondents refer to Table 1.

**Table 1:** Socio-Demographic Information of Respondents

Feature	Sample Details		Feature	Sample Details	
	Older Indians living with adult children (15)	Older Indians living without their adult children (15)		Adult children living with older parents (15)	Adult children not living with older parents (15)
Mean age	Mean: 74.6 Std dev: 8.04 Age Range: 65–80	Mean: 74.1 Std dev: 7.87 Age Range: 65–80	Mean Age	Mean: 37.9 Std dev: 6.13 Age Range: 30–45	Mean: 37.5 Std dev: 6.12 Age Range: 30–45
Gender	Older males: 53% Older females: 47% Older males: 8/15 Older females: 7/15	Older males: 47% Older females: 53% Older males: 7/15 Older females: 8/15	Gender	Adult male child: 40% Adult female child: 60% Adult male child: 6/15 Adult female child: 9/15	Adult male child: 67% Adult female child: 33% Adult male child: 10/15 Adult female child: 5/15
Religion	Hindus: 67% Jains: 20% Christians: 13% Hindus: 10/15 Jains: 3/15 Christians: 2/15	Hindus: 53% Jains: 27% Christians: 20% Hindus: 8/15 Jains: 4/15 Christians: 3/15	Occupation	Corporate Professionals: 73% Studying: 13% Lawyer: 7% Journalist: 7% Corporate Professionals: 11/15 Studying: 2/15 Lawyer: 1/15 Journalist: 1/15	Corporate Professionals: 53.33% Studying: 33.33% Lawyer: 13.33% Corporate Professionals: 8/15 Studying: 5/15 Lawyer: 2/15
Source of income	Still working: 53% Part time working: 20% Pension: 27% Still working: 8/15 Pension: 4/15 Part time working: 3/15	Still working: 47% Part time working: 27% Pension: 27% Still working: 7/15 Pension: 4/15 Part time working: 4/15	Level of Education	Post-Graduation:87% Pursuing PhD: 13% Post-Graduation:13/15 Pursuing PhD: 2/15	Post-Graduation: 67% Pursuing PhD: 33% Post-Graduation: 10/15 Pursuing PhD: 5/15
Level of education	PhD: 60% Post-Graduate: 27% Under-Graduate: 13% PhD: 9/15 Post-Graduate: 4/15 Under-Graduate: 2/15	PhD: 67% Post-Graduate: 27% Under-Graduate: 7% PhD: 10/15 Post-Graduate: 4/15 Under-Graduate: 1/15	Marital status	Married: 53% Unmarried: 47% Married: 8/15 Unmarried: 7/15	Married: 67% Unmarried: 33% Married: 10/15 Unmarried: 5/15
Ethnic groups	Tamils: 33.3% Bengalis: 33.3% Punjabis: 33.3% Tamils: 5/15 Bengalis: 5/15 Punjabis: 5/15	Malayalees: 27% Gujaratis: 40% Telugus: 33% Malayalees: 4/15 Gujaratis: 6/15 older male respondents and 3/15 Telegus: 5/15			
Living arrangement	With spouse, adult child, daughter in law and grandchild/children: 40% With spouse and unmarried adult child: 47% Widowed and living with unmarried adult child: 7% Widowed and living with adult child, daughter in law and grandchild/children:7% With spouse, adult child, daughter in law and grandchild/children: 6/15 With spouse and unmarried adult child: 7/15 widowed and living with unmarried adult child: 2/15	With spouse: 60% Widowed: 40% With spouse: 9/15 Widowed: 6/15			
Total number of children	Two children: 40% One child: 60% Two children: 6/15 One child: 9/15	Two children: 60% One child: 40% Two children: 9/15 One child: 6/15			

Post the data collection, the interviews were analyzed through thematic analysis (Creswell, 2013). The responses were broken into codes, and from those codes, the major themes were identified as the findings of the study. Thus, while several important outcomes emerged, the ones which indicated a pattern among all the responses, have been included in the findings section.

## Methodological Rigour

Establishing methodological rigour is an important responsibility of scholars across disciplines using any form of research method. In a recent article, Phoenix (2018) suggests that the concept of rigour has evolved significantly in recent times in the discipline of gerontology. Studies with proper parameters of measuring reliability and validity are considered to be methodologically robust studies. However, scholars of the qualitative paradigm have suggested that factors such as reliability and validity are mostly associated with quantitative studies and do not pertain to qualitative studies (Cypress, 2017; Leung, 2015; Morse et al, 2002). Hence, several scholars of qualitative research (Connelly, 2016; Cope, 2014; Oliver, 2011; Thomas & Magilvy, 2011) use trustworthiness containing four features: credibility, transferability, dependability and conformability, the substitute of reliability and validity as suggested by Lincoln and Guba (1985) in their seminal work to establish the vigour of their qualitative research. Relatedly, scholars of gerontology (Buescher and Grando, 2009; Koenig et al, 2011; Cypress, 2017), have also used this four criteria of rigour to demonstrate methodological rigour in their qualitative research.

Similar to these studies, the present study also attained methodological rigour, by following the four criteria of rigour as indicated by Lincoln and Guba (1985).

**Credibility:** This feature measures the accuracy of the results (Lincoln & Guba, 1985). As indicated in the Methodology Section, to ensure the authenticity of interviews face to face interviews were conducted with all the older Indians and their co-resident adult children. Additionally, the interviews for the non-resident adult children, which were administered through video calls, were audio-recorded. Only one older adult and their co-resident or the non-resident adult child were interviewed per day. After every interview, to avoid missing out on important information, the interviews as well as the field notes, were transcribed verbatim. Additionally, the transcribed data and the final themes of the study were also cross-checked with some of the study respondents.

**Transferability:** This feature denotes the applicability of the research design or study findings in different contexts. Usually, thick description is used to establish transferability in the study (Lincoln & Guba, 1985). By providing a detailed description of the study setting, data collection and processes of investigation and analysis, transferability was ensured for the present study. The interviews for the present study were stopped only after the data was saturated and repetitive. In the course of analysing the data, the transcribed interviews were read multiple times to be sure of the themes. Next, the responses of both the groups (older adults as well as the adult children) were compared to identify the themes of the study. Initially, all the possible themes were listed and documented. Later those themes which could not be linked to all the participant responses were removed. Finally, those themes which were common in the narratives of all the respondents were considered to be the main findings of the study.

**Dependability:** This feature refers to accomplishing similar results with other groups in the same study setting (Lincoln & Guba, 1985). To validate the study findings, the author reached out to non-respondents with similar experiences (other older Indians growing old in Saskatoon) and conducted similar interviews among them. Following their interviews, the author compared the main themes emerging from the interviews of the respondents and the non-respondents. Since similar patterns were recognized in the responses of the respondents and the non-respondents, the themes observed from the responses of the respondents were finalized for the present study.

**Conformability:** This feature requires the author to maintain an audit trail for other researchers to review and approve that the author’s findings are logical (Lincoln & Guba, 1985). An audit trail consisting of the author’s field notes, audio recordings, time diaries and reflexive notes were maintained to guarantee conformability of the present study.

## Main Findings

### Physical Disabilities and Perceptions of Ageing in a Transnational Setting

The interviews revealed that all the older adults were undergoing some form of bodily decline. Muscle pain, arthritis, cardiovascular issues, cataract and high blood pressure, were some of the most common health issues cited by these older adults. In particular, the older respondents stated that they did not put much effort into being fit and active as they were aware of their disabilities. For instance, as the quotes of a few older respondents elucidate:

*“I am aware of one fact that I am 70. No matter how much I remain healthy and fit, I do not mind admitting that I do get tired. I do have some body pains as well in case of too much physical exertion. Here in Saskatoon, frailty and dependence are considered to be failures. You have to constantly stay fit and energetic. But I honestly do not care. I do not exert myself overtly.” (Older male respondent, aged 70).*

*“I have several problems with my body. I have back pain and my various joint pains as well. Additionally, I do not sleep well and have to go to the bathroom several times as well. Compared to Western standards, I am not that old. Back in India, I could have easily hired someone to do my work. But here I still have to perform the household chores because labour is very expensive here. So I do only what is needed to do for my daily living such as cooking and cleaning.” (Older female respondent, aged 67).*

*“Two years ago I was diagnosed with severe cataract. Despite a successful surgery, I still have some vision problems. As a result, I cannot take part in many activities and also have restricted my everyday mobility. But I have no regrets.” (Older male respondent, aged 65).*

These accounts suggest that in spite of growing old in a transnational context such as Saskatoon, the older Indians accepted their various physical limitations and health issues. In their perception of growing old, the older adults recognized their dependence, weakness and fragility as well as acknowledged the pointlessness of being fit and energetic in their later lives.

### Intergenerational Relationships and Caregiving Arrangements

The findings of the study showed that older adults from both groups (Group I and Group II), did not hinder the life choices of their adult children. Older adults from both groups indicated that their adult children irrespective of their gender made independent decisions with regard to their higher education, jobs, marriage, grandchildren’s education and financial investments. The older adults from both groups also specified that they did not advise their adult children on their daily lifestyle either. Despite the lack of interference on the major decisions or in the daily activities, older adults from both groups expected their adult children to look after them in case of a medical exigency:

#### Group I

Quotes from Older Indians who live with their adult children and quotes of those adult children as well:

*“My wife and I live with our adult son. Unlike other children who left home at the age of 16, my son is still here with us. He is working here, and so I don’t see the point of his shifting to another apartment. Even though we live together, we do not interfere in each other’s space. In fact, even major decisions such as marriage and job change, we do not interfere. He does not help in our daily chores but, we know if anything major happens like a health emergency, he will do everything.” (Older male respondent, aged 70).*

*“I never left home. Most children here start living independently at the age of 16. But I have been living with my parents forever. I think it works out well for both of us. They are pretty liberal, and have given me my space and so I do not have a problem living with them. But in case of a major problem, we are there for each other. Also, I have told them that no matter what, if they become disabled or terminally ill, they will have to live with me wherever I am.” (Adult male child, aged 32).*

*“Our son has been living with us forever. He did not leave Saskatoon, and even after his marriage, he stayed with us. It will be wrong to say that we do not have conflicts. I do have everyday issues with my daughter in law. Simple things like what to cook and childcare-related issues. But overall, we strive to maintain peace. However, since my husband and I do not interfere in their major decisions like our grandchild’s education and their financial investments, they continue to live with us. We as parents give them sufficient space, and we do not accompany them in every outing or trip either. We let them be.” (Older female respondent, aged 74).*

*“I think it is good that we live with our parents. I never wanted my child to be in daycare or crèche. That was one of our major reasons to live in Saskatoon. I have received many offers of working from Toronto and Ontario, but I did not change because of our child. Plus my parents do not restrict our lives. All the major choices, I consult my wife and we take a joint decision. Also, I help my parents in paying their bills and buying their grocery. Plus in case of a medical situation, I can rush them to the hospital. I will look after them and provide care to them” (Adult male child, aged 37).*

*“I live with my daughter. My son is married and settled in the USA. Since my daughter is a practising doctor in the hospital, she lives with me. We often have arguments. Small things like she never cleans her room and never switches off the television. But she is also my support system. We also do a lot of things together, like shopping, going to the cinema and weekend trips. Other parents would have been worried about her marriage but, I am happy with this arrangement and so I never even ask her about marriage.” (Older female respondent, aged 69).*

*“I share everything with my mom. Whom I am dating, what happens at my workplace, everything. We are very close. Here in the West, there is no pressure to get married. So I am happy that I have crossed thirty and still I do not feel the need to get married. Of course, sometimes I do feel insecure that what will happen if mother passes away all of a sudden, but then I do not get married just for the sake of marriage. I am happy in this space, and my mother also does not compel me for marriage at all. Hence, this is a conducive arrangement for both of us. However, the future is uncertain. I might get married and, I am worried who will look after her. But one thing I have made clear that if she loses her mental and physical health completely, she will have to come and live with me.” (Female adult child, aged 33).*

Most of the non-cohabiting adult children had migrated to the USA or in other cities of Canada for better employment or educational opportunities. Though they did not reside with their parents, nonetheless, they were in constant touch with them through phone or video calls. Similar to older parents in Group I, older parents who did not live with their adult children (Group II) gave space to the major and minor choices of their adult children. Additionally, older adults in Group II were not keen on relocating with their adult children as they were more engaged and connected in Saskatoon. However, older parents in Group II also expected that their children would arrive immediately during a health emergency:

#### Group II

Quotes from Older Indians who do not live with their adult children and quotes of those adult children as well:

*“Both our children left home early on. They studied outside Saskatoon and are now settled in the USA. We visit each other as and when we can. We speak to both our children at least once every day. However, both my wife and I know that both our children will rush to Saskatoon in case anything happens to either of us. My daughter in law is a Canadian. Initially, we were a bit sceptical about their match because her culture is completely different, but then we gave our consent. So we respected his choice, and now we have two lovely grandchildren as well.” (Older male respondent, aged 71).*

*“There are very few opportunities in Saskatoon. Most children move out of Saskatoon for studying and working purposes. I also did the same. We try to visit our parents as much as we can but then children also want to visit tourist spots during their vacation. I do speak to my parents at least once a day and ask about their health and other issues. But I think living separately is a better arrangement for both of us. We get space and privacy. Of course, in case of a health emergency, I will rush to their side, and in case one of them is disabled, I will have to make arrangements to bring them here and live with me. But for now, the distance works fine.” (Adult male child, aged 42).*

*“My daughter lives in the USA. I do go and visit her from time to time, and she also comes, but I like it here in Saskatoon. I am actively involved in community gardening and also teach part-time at the nearby school. I feel lost in California. My daughter will rush and come if I have a heart attack. So although my daughter and I live alone and I can easily shift in with her, I prefer to live separately. This way, we both get our respective privacy and space.” (Older female respondent, aged 74).*

*“I think it is better that my mother and I live separately. I am worried about her, and we do talk on the phone every day, but I know she is happier in Saskatoon. I respect her choice, and I think she is more involved in Saskatoon. Here when she used to live with me, I had to keep her busy the whole day. So this way both of us are happy in our respective spaces. Though I have told her that I will bring her to the USA in case she loses her physical and mental health completely.” (Adult female child, aged 35).*

Comparing the quotes, it may be suggested that older adults from both groups believed in giving individual space and freedom to their adult children. In both groups, the older adult shed their roles as kin keepers (Lamb, 2009, 2013; Gangopadhyay & Samanta, 2017) and did not influence the life choices of their adult children. In particular, older adults from both groups took organizing roles in several of the local Indian associations and also participated in most of the community-oriented activities. Though these older adults had their network ties for everyday engagement, nonetheless, for critical health issues, they expected caregiving support from their adult children. Likewise, the adult children also emphasized on providing physical, mental and financial support to their older parents during permanent disability or terminal illness. Specifically, the non-resident adult children insisted that they would rush immediately to Saskatoon in a situation of a health crisis. Non-resident adult children also emphasized that while they might have to rely on paid care to look after their parents, nonetheless, they would visit more often or use home cameras to monitor the activities of the hired help. Expectations of filial obligations and later life emotional and physical support were similar from adult sons and daughters.

### Later Life Gender Roles, Widowhood and the Transnational Setting

The narratives of the respondents suggested that Indian older women enjoy more mobility and parity in Saskatoon, as opposed to older women in India. Additionally, the Indian older widows in Saskatoon indicated that they are far less constrained and lead much more independent lives in Saskatoon. These widows irrespective of their religious background mentioned that they do not abide by any restrictions and have more control over their lives. Apart from enjoying more freedom, the responses of the widows as well as the older women also indicated that they have their group of friends for hanging out and going on road trips. Through the quotes (indicated below), the present study demonstrates how these older women construct their gender roles in a transnational setting:

*“I never felt like a widow. Even after my husband’s death, I did not do things any differently. I never stopped dressing up or eating any food. So I never felt any less confident or invoked sympathy being a widow. In fact, even as a widow, I worked and supported my child. Here it is not like India, where an old woman is a burden. Here in Saskatoon, we have more equality and freedom do to things” (Older female respondent, aged 76).*

*“Ever since I came to Saskatoon, we have divided our housework. Sometimes he cooks, sometimes I do. In fact, when I am unwell, he does all the housework. So just because I am a woman, I have never felt that I should do all the household chores. We are both doctors, and so I do not see why I am any less than my husband.” (Older female respondent, aged 68).*

Not only older women but older men also believe in dividing housework and emancipating their wives. In particular, the older respondents (both men and women), suggested that due to high labour costs in Saskatoon, they practised equal distribution of household work since their initial days of immigration.

*“In Saskatoon, older men and women are the same entities. There is no discrimination. My wife and I do the same activities and also divide all our housework. It is not like India, where men feel ashamed to do housework. Here it is a matter of pride.”(Older male respondent, aged 74).*

*“I actually encourage my wife to dress more and go out with her friends. Here it is not like India, where women are at home and dependent on their husbands. They have their own groups, and they also organize several events. My wife also won a few awards in community welfare.” (Older male respondent, aged 66).*

Based on these quotes, it may be suggested that the transnational setting enables Indian older women (widowed and married) to engage in various activities and events in their later lives. Additionally, in the transnational setting, the older men also provide support by sharing household chores.

## Discussion

The findings suggest that older Indians settled in Saskatoon are conscious of their physical ailments and do not follow any particular fitness regimes. Acknowledging their old age, the older adults avoided excess mobility and physical exertion. Despite the predominance of the Successful Ageing movement in North America (Havighurst, 1961; Lamb, 2014, 2017; Moody, 2009; Rowe & Kahn, 1997; Zacher, 2015), the narratives of the older Indians settled in Saskatoon indicated an alternate approach to ageing. Gerontological studies in India have demonstrated that older Indians associate physical decline with old age and are not embarrassed to admit their health problems (Vatuk, 1990; Van-Willigen & Chadha, 1999; Lamb, 2002, 2009; Gangopadhyay, 2017; Ahlin, 2018). Corroborating with these studies on older adults in India, the present study found that the perceptions of ageing among older Indians ageing in Saskatoon was similar to their Indian counterparts. Despite immigrating at a young age and growing old in a transnational setting, the older Indians deny the Successful Ageing ideal and their physical weaknesses constitute an important component in their perceptions of ageing.

Similarly, with regard to intergenerational relationships, while the older Indians believed in giving liberty to their adult children in their everyday lifestyles as well as in taking independent decisions relating to marriage, jobs and grandchildren’s education, nonetheless, they expected their adult children to fulfil filial obligations in case of an emergency health condition. Studies on intergenerational relationships among financially stable older adults in India (Bhat & Dhruvarajan, 2001; Lamb, 2009; Gangopadhyay & Samanta, 2017; Ahlin, 2018), have highlighted that older parents expect emotional and physical caregiving arrangements from their adult children. Similar to these studies on intergenerational relationships in India and, the present study illustrates that older Indians settled in Saskatoon continue to expect caregiving support from their adult children during a critical health issue. However, in contrast to studies on caregiving arrangements in India which suggest that older parents expect mental and corporal support from adult sons and not adult daughters (Vatuk, 1990; Vera-Sanso, 2005, 2007; Lamb, 2009; Dhar, 2012), the present study found that older Indians settled in Saskatoon kept similar expectations from both their adult sons as well as daughter.

Finally, the study also found that gender roles in later lives are blurred in a transnational context. Several studies have highlighted the various forms of oppression faced by older women in India. In particular, these studies have noted how irrespective of their religious community, older women, especially widows are expected to embrace ageing as per the patriarchal structures of Indian society (Vatuk, 1990; James, 1994; Cohen, 1998; Lamb, 1997, 2002; Brijnath & Manderson, 2008; Brijnath, 2014; Gangopadhyay & Samanta, 2017). As per Hindu and Jain traditions, older widows are expected to wear white, abstain from any food that might generate heat in the body and spend their later lives dedicated to religion and spiritual activities (Chen & Dreze, 1992; Lamb, 2002; Jensen, 2005; Vera-Sanso, 2005; Brijnath, 2014; Gangopadhyay, 2017). On the other hand, older Muslim and Christian women in India have to deal with economic and social deprivation. Older Muslim and Christian widows have to depend on unions or religious bodies such as Roza (associated with the Mosque) or Church communities to receive later life health and economic support (Rajan, 1999; James, 1994; Gangopadhyay, 2017). Additionally, middle class older women in India, post widowhood, restrict themselves to the domestic and fulfil grandparenting duties (Gangopadhyay, 2017; Lamb, 2009). In contrast to older women in India, their counterparts in transnational contexts experience more gender equality and participate in activities beyond the realm of their household (Kalavar & Van-Willigen, 2005; Lamb, 2014; Sudha, 2014). The study departs from the existing studies on older women and widows in India (Vatuk, 1990; James, 1994; Cohen, 1998; Lamb, 1997, 2002; Brijnath & Manderson, 2008; Brijnath, 2014; Gangopadhyay & Samanta, 2017) and suggests that older women, including widows, have more independence in terms of their mobility and daily life in a transnational setting.

Lack of research on South Asians, particularly Indians ageing in transnational settings is one of the research gaps in gerontological research. By providing evidence on ageing experiences, intergenerational relationships, and gender ideologies in later lives, the main aim of the present study was to address this research gap in gerontological scholarship.

## Concluding Thoughts

The main aim of this paper was to examine the perceptions of ageing, intergenerational relationships and gender roles among Indian older adults who have grown old in Saskatoon. Through its findings, the paper found that these older adults have resisted the Successful Ageing paradigm by accepting their bodily disorders. A comparison of older adults living with their adult children and older adults not living with their adult children showed that individual privacy, space and freedom are integral components in shaping intergenerational relationships among older Indians and their families. However, in spite of individual freedom being maintained between the older parent/s and the adult child, filial obligations gained prominence with regard to later life care. Finally, the paper also investigated gender roles among these older Indians and found that older women, including widows, lead restriction-free lives and older men and women, contribute equally to household chores.

The strength of the study lied in the sample population of the study. Prior studies on older Indians have focussed on older adults who have immigrated to transnational settings as senior citizens (Lamb, 2009; Lunt, 2009; Patel, 2011). On the other hand, the present study has examined the perceptions of ageing and older parent and adult child relationship among older Indians settled in Saskatoon. The other strength of the study is that it is located in Saskatoon. Saskatoon as a study site is less explored by gerontological scholars, and this study provides evidence on the intersections between care arrangements, constructions of growing old and gender roles in one of the potential emerging economic cities of Saskatchewan, Canada. However, the study also has a few limitations. As the study had relied on snowball sampling, most of the older respondents were Hindus and Jains. Among the older respondents, only five belonged to the Christian community. Additionally, the older respondents belonged to the upper-middle and middle-class category. Thus the findings from this study cannot be generalized as they are representative of a very select segment of the India immigrant population. Also, due to the limited amount of time for the interviews, the study could not document the impact of State policies and economic changes on shaping perceptions of ageing and intergenerational relationships. Despite its limitations, the study does add to the growing research on intergenerational care, ageing and transnationalism as it examines the older Indian community in Saskatoon.

To expand the gerontological scholarship on minority communities, future studies should compare the experience of growing old among different South Asian communities ageing in transnational settings.
